# Supplementary material for: Real-space imaging of a phenyl group migration reaction on metal surfaces
Source: Nat Commun. 2023 Feb 21;14:970. doi: 10.1038/s41467-023-36696-6 (PMC9944283; doi:10.1038/s41467-023-36696-6)
Supplement: Supplementary file 1 — Supplementary Information [file 41467_2023_36696_MOESM1_ESM.pdf]

Supplementary Information for

Real-Space Imaging of a Phenyl Group Migration Reaction on Metal Surfaces

Authors: Zilin Ruan[1, #], Baijin Li[1, #], Jianchen Lu[1, #, *] Lei Gao[2, #, *], Shijie Sun[1], Yong Zhang[1], Jinming Cai[1, *]

Affiliations:

[1]Faculty of Materials Science and Engineering, Kunming University of Science and Technology, Kunming, Yunnan 650093, P.R. China

[2]Faculty of Science, Kunming University of Science and Technology, Kunming, Yunnan 650500, P.R. China

This file includes:

Supplementary text

Supplementary figures 1-30

Supplementary table 1-6

Supplementary references 1-6

Supplementary text

Precursor purification. DMTPB was purchased from Sigma Aldrich, and further purified in a home-made MBE chamber with a standard Knudsen evaporator monitored by an SQM-160 Rate/Thickness monitor.

STS measurement. dI/dV measurements have been performed with a lock-in amplifier (Zurich Instrument HF2LI) with a modulation bias of V_{rms}= 20 mV and f = 599 Hz.

Mechanism of high-resolution bond-resolved scanning tunneling microscopy. Bond-resolved STM (BR-STM) allows for sub-molecular resolution imaging of various as-synthesized species, which is useful for understanding internal reaction mechanisms. The constant-height BR-STM imaging with a CO decorated tip is conducted in the Pauli repulsion regime, where the CO molecule attached to the tip-apex undergoes a lateral bending with the largest deflection magnitude over the electron-rich areas presented by chemical bonds. Such a deflection of the CO molecule effectively modulates the overall tunneling conductance of the tip−sample junction, leading to the appearance of sharp line features over the positions of chemical bonds in tunneling current images, and has become a powerful tool to elucidate on-surface chemical species.

Supplementary Figures 1-30, Supplementary Tables 1 and 6.

1. Proposed chemical models of the DMTPB self-assembly on Au(111) surface.

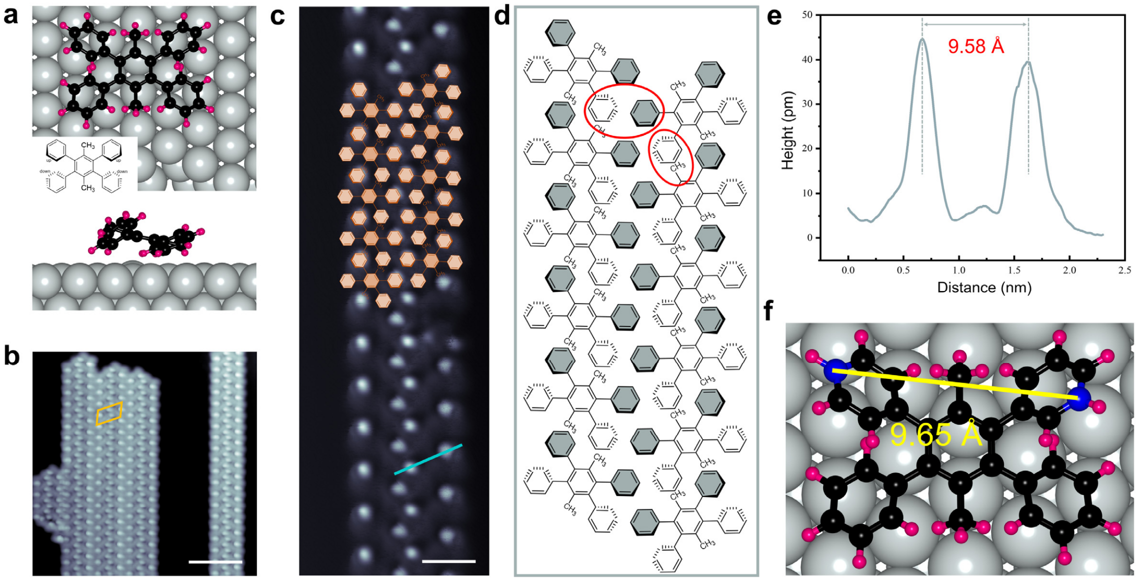

Supplementary Figure 1. Identification of a DMTPB monomer in the self-assembly on the Au(111) substrate. a Front and side views of optimized adsorption configuration of a DMTPB monomer on Au(111) substrate. Two up-tilted benzene rings due to steric hindrance are depicted in the inset. **b** Constant-current STM topography (I = 30 pA, V_s = 1 V) of DMTPB self-assembly. **c** Constant height STM image (V_s = 1V) of the linear self-assembly with partially overlaid chemical models. **d** The corresponding chemical structure models of the self-assembly are stabilized by the interactions between the benzene rings and methyl groups, namely, π-π and –CH··· π interactions, as marked by the red ellipse. **e** A line profile measured between two bright dots in (**a**), as denoted by the blue line in (**c**), where the derived distance is 9.58 Å. **f** The measured distance between two carbon atoms in the two up-tilted phenyl groups (see also supplementary Fig. 2, P1 and P2) based on DFT optimized model. Scale bar: (**b**) 5 nm; (**c**) 1 nm.

2. Surface adsorption configuration of precursor DMTPB on Au (111) surface.

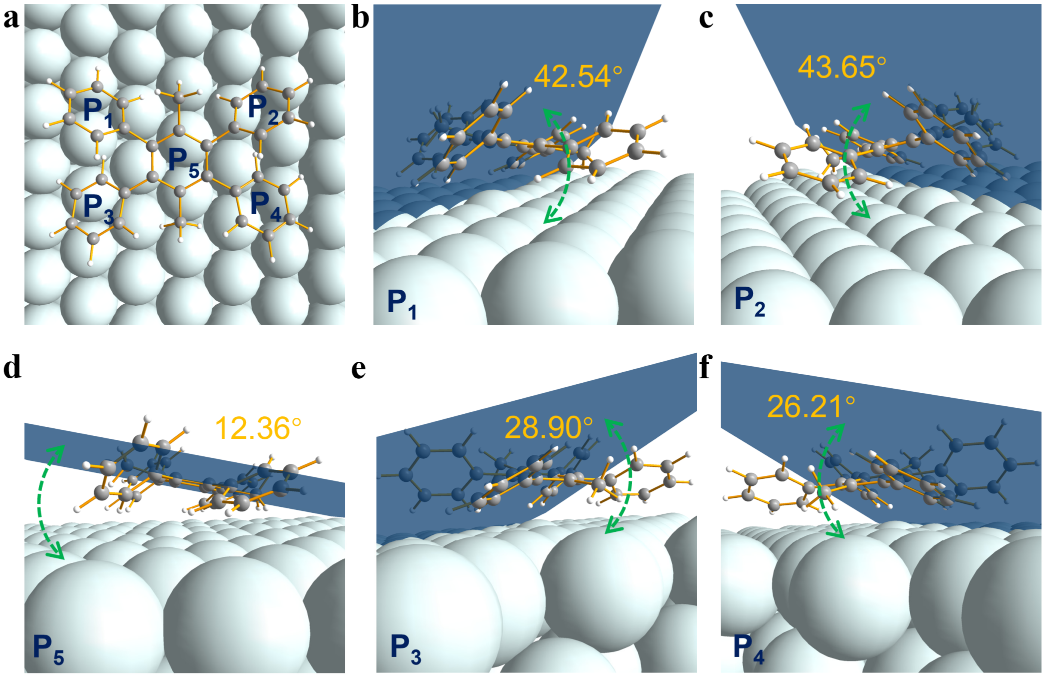

Supplementary Figure 2. Relative angles between five benzene rings of the DMTPB molecule and the Au(111) surface. a Top view of a DMTPB monomer on Au(111). The five benzene rings are numbered by P_1-P_5, respectively. **b-f** Angles between the different benzene ring planes and the Au(111) substrate. Note that the dehydrogenation begins at the methyl group which is closer to the substrate.

3. Early-stage on-surface products of the DMTPB on the Au(111) substrate after annealing at 440 K.

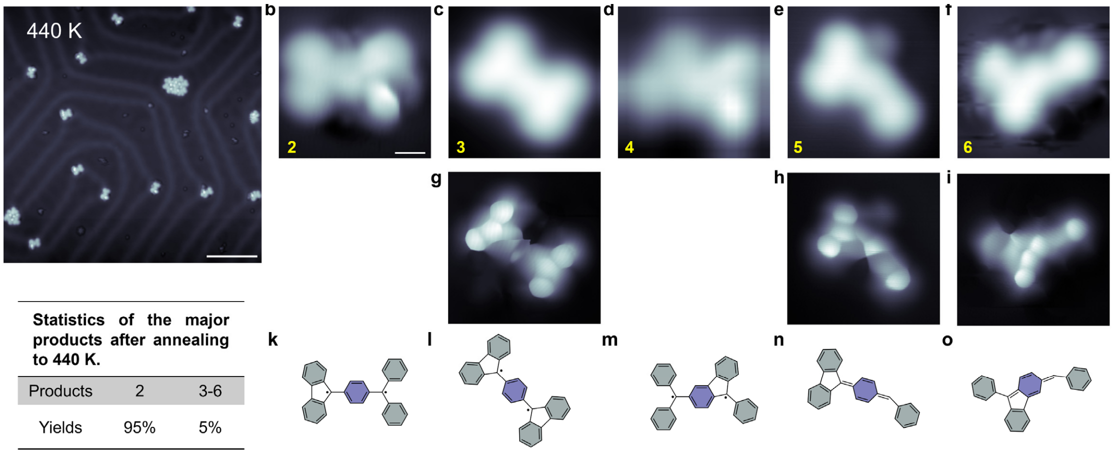

Statistics of the major products after annealing to 440 K.

Products	2	3-6
Yields	95%	5%

Supplementary Figure 3. STM topographies of intermediates obtained at the early annealing process. a Large-scale STM topography after annealing at 440 K. **b-f** Different monomers observed on the surface. **g-i** The corresponding BR-STM topographies of monomer **3**, **5** and **6**. **j** The statistic of the monomers at 440 K. **k-o** The corresponding chemical models of **7-11**. Scanning parameters: (**a**) I = 50 pA, V_s = 200 mV; (**b-f**) I = 50 pA, V_s = 100 mV; (**g-i**) V_s = 2 mV. Scale bar: (a) 8 nm; (**b-i**) 0.5 nm.

4. **Supplementary Table 1. Statistics of the monomer products after annealing the DMTPB self-assembly on Au(111) to 590 K**

Supplementary table 1: Statistics of the monomer products after annealing the DMTPB self-assembly on Au(111) to 590 K.

Products	A1	A2	A3	A4	A5	
Counts	78	30	13	14	51	186
Yield	42%	16%	7%	8%	27%	100%

The high yield of **A1** at 590 K suggested a structural rearrangement of intermediate **2** at higher temperatures since it cannot be formed directly from the dominating intermediate **2**. Considering the fact that species **8, 9** and **11** (Supplementary Fig. 10) are also observed at a slightly higher temperature (445 K-465 K), a C-C bond cleavage between the two migrated phenyl groups and subsequent ring closure between the phenyl group and the central benzene ring is expected and accounts for the unusually high yield of **A1**. Additionally, **A1** can be obtained from specie **4**, the desorption of other species during the annealing process could potentially lead to an increased proportion of **A1** at 590 K. However, the relatively low yield of **4** would not result in such a high yield of **A1** at 490 K, a ring opening of the fluorene group thus prevails.

5. Additional topographic images of products A1, A2, A3 and A4.

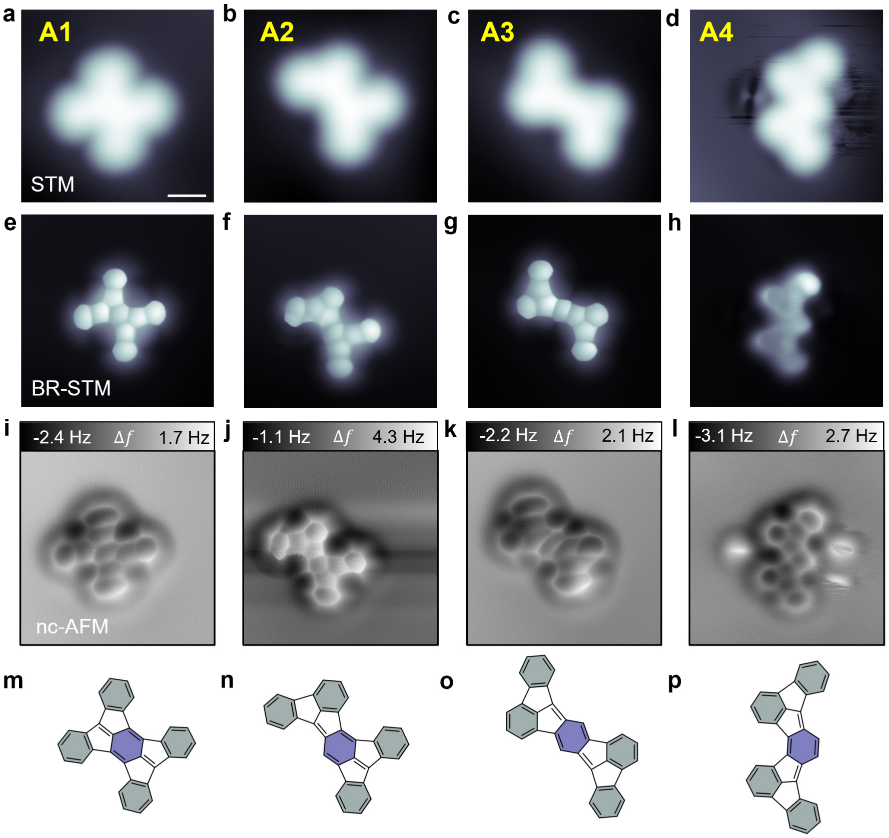

Supplementary Figure 4. Additional High-resolution STM and nc-AFM images of final PAH products A1, A2, A3 and A4. a–d Constant current STM topographies (CO tip, I = 50 pA, V_s = 100 mV) of the products **A1**, **A2**, **A3** and **A4**, respectively. **e–h** BR-STM images, **i–l** nc-AFM images (CO tip, V_s = 2 mV) and (**m-p**) Identified molecular structures corresponding to panels (**a–d**), respectively. Scale bar: 0.5 nm for all topographic images.

6. Surface-induced chirality.

In addition to the real-space imaging of reaction products, the submolecular resolution capability of BR-STM also allows us to identify the chirality of reaction products in large-scale imaging. Interestingly, products **A3** and **A5** are achiral in the gas phase but show a surface-absorption-induced chirality. The enantiomers of product **A5** can transform from one to another through the flip of the fluoranthene motif in the gas phase. However, this is not likely to occur on the surface because of a high energy barrier arising from the large size of these molecular motifs, which is also responsible for the presence of not fully

cyclodehydrogenated product **A5**. Therefore, the two enantiomers of product **A5** are most likely to derive from the symmetrical dehydrogenation process from intermediate 1d.

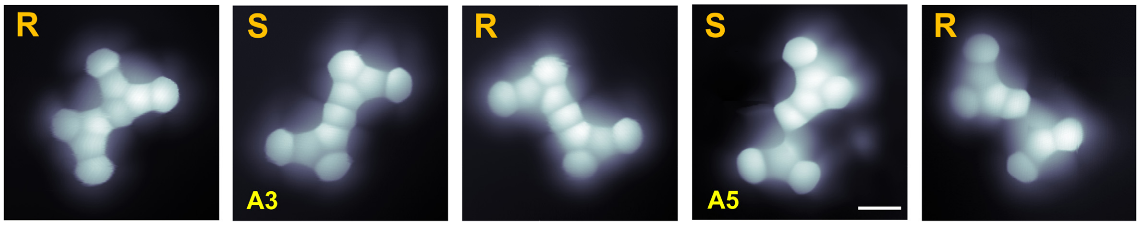

Supplementary Figure 5 | Surface-induced chirality. BR-STM images of products **A2**, **A3** and **A5** show a surface-induced chirality (CO tip, $V_s = 2$ mV). Scale bar: 0.5 nm.

7. Electronic properties of the fully cyclodehydrogenation products.

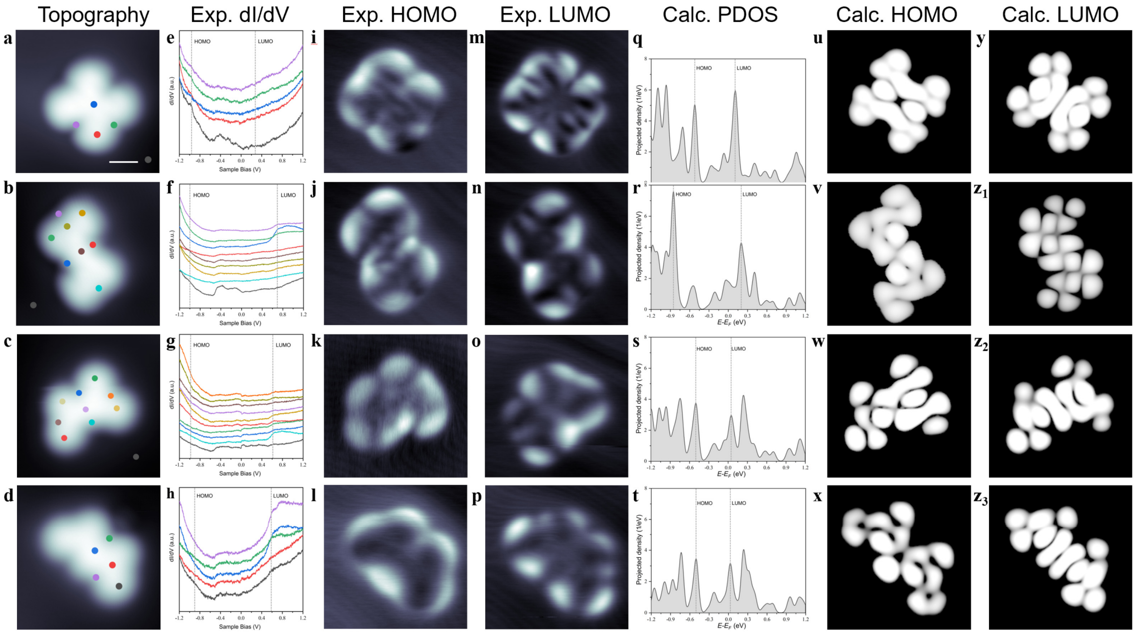

Supplementary Figure 6. Electronic properties of fully cyclodehydrogenated PAH products A1, A2, A3 and A4. a-d STM topographies, **e-h** dI/dV point curves, **i-l** Experimental HOMO and **m-p** LUMO, **q-t** Calculated PDOS, **u-x** HOMO and **y-z_3** LUMO for products **A1**, **A2**, **A3** and **A4**, respectively. dI/dV curves taken at positions in (**a-d**) are denoted with the same-colored dots. Scale bar: **(a-d)** 0.6 nm, **(i-p)** 0.5 nm.

8. High-resolution characterization of product 2.

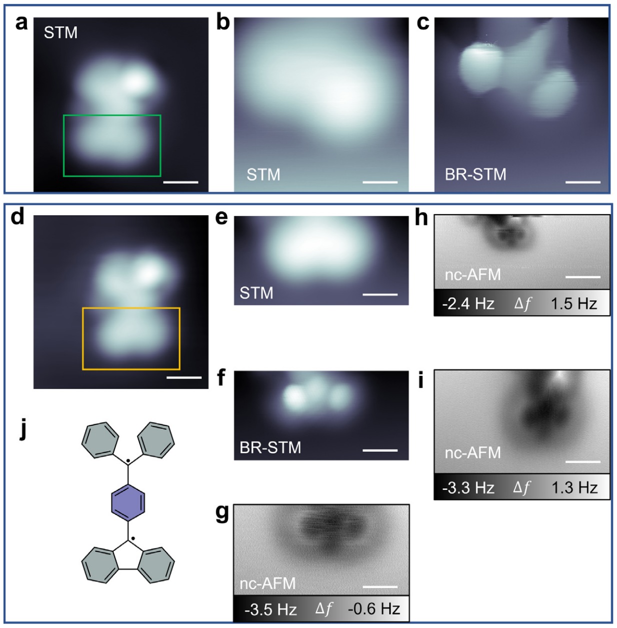

Supplementary Figure 7. High-resolution characterization of product 2. a-c Constant current STM image, zoom-in STM and BR-STM image of the planar part of product **2**, respectively. **d-e** STM, **f** BR-STM and **g-i** nc-AFM images of the planar part of product **2**. **j** Diradical form of product **2**. Note that **a-c** and **d-h** are taken from two different monomers. A gentle distortion in the nc-AFM image in **h** is caused by drift. Scanning parameters: (**a**, **b**, **d, e**) I = 50 pA, V_s = 100 mV; (**c**, **f-i**) V_s = 2 mV. Scale bar: (a) 6 Å; (**b-c**) 2.4 Å; (d-f) 4 Å; (g) 9 Å; (h) 5 Å.

9. Structural assignment of product 2.

A comprehensive comparison of the experimental nc-AFM images and simulated nc-AFM images indicates that product **2** is in the diradical form, which does not contain the hydrogen atoms at the sp3 center of the apex of the five-membered ring. In the simulated nc-AFM images of the diradical form for product **2**, the twisted phenyl rings are well reproduced, as indicated by orange, green and red arrows in Supplementary Figs. 8f, 8k and 8l. while such features are absent in the H saturated form. Moreover, the hydrogen atoms in the sp3 center give pronounced features (Supplementary Figs. 8o-q, white arrow) which are absent in the experimental nc-AFM images (Supplementary Figs. 8c-f). Although the features of the twisted phenyl groups are also visible and hydrogens pointing out the surface disappear at a

very close tip-sample separation (Supplementary Figs. 8r), this would lead to a distortion of the molecule and a considerable background signal, which is strikingly different from our experiments. We also rule out the possibility of the quinoid form of product **2**, since this would lead to a planar configuration (Supplementary Figs. 9g-9i) and contradict the observation in Supplementary Figs. 8c-f, where the nonplanar feature is identified. In conclusion, we assign product **2** to the diradical form.

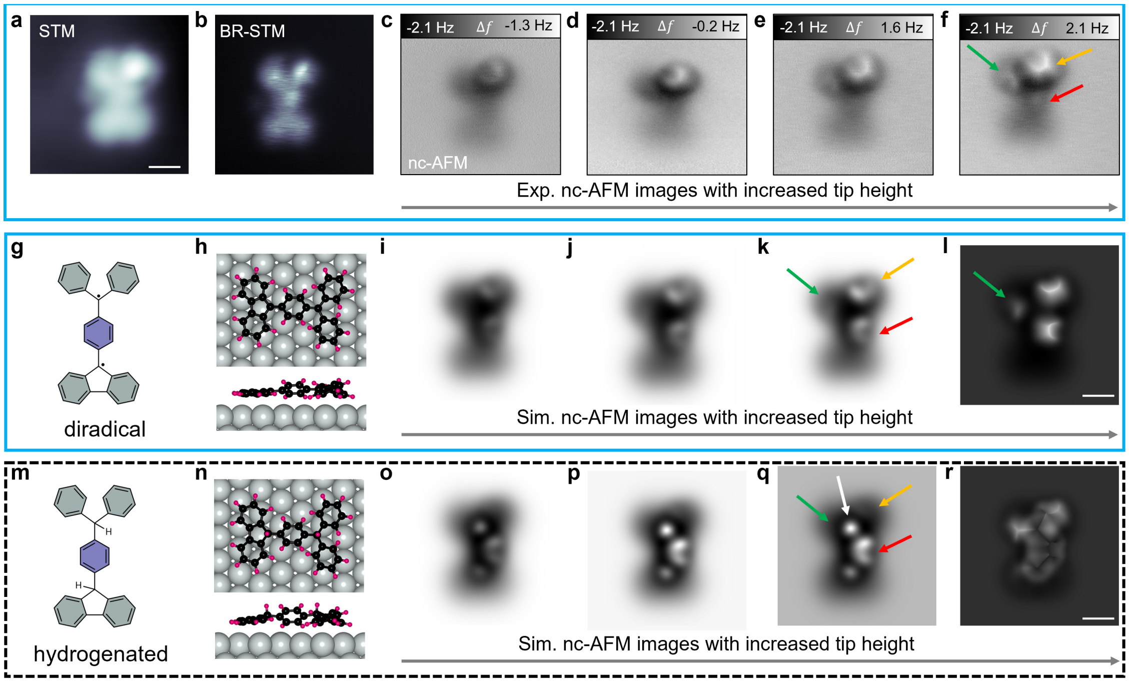

Supplementary Figure 8. Structural elucidation of product 2. a Experimental STM, **b** BR-STM and **c-f** nc-AFM images of product **2**. **g, m** Chemical structures, **h**, **n** optimized adsorption configurations and **i-l o-r** simulated nc-AFM images of product **2** without and with H saturation. Scale bar: 0.6 nm for all topographic images.

10. Structural assignment of product 3.

Our comprehensive nc-AFM simulations of three possible chemical structures, i.e., hydrogen atoms saturated form, canonical diradical, or quinoid structures, indicate that product **3** is in the form of diradical hydrocarbon, as can be judged from a comparison of the experiment results and simulations in Supplementary Fig. 9, as follows:

(i). In the experimental nc-AFM images obtained at a moderate tip-sample separation, the

outer edges (denoted by yellow arrows in Supplementary Fig. 9) of the fluorenes are well resolved, which coincides with the simulated nc-AFM image (Supplementary Fig. 9g), while such features are absent in the saturated form in Supplementary Fig. 9c. At a relatively close tip-sample separation, although the edges are also identified for the hydrogen saturated form, the fluorene groups are distorted, which is not in accordance with the experimental results.

(ii). The apex of the five-membered rings (denoted by the orange arrow in Supplementary Fig. 9) is well resolved in the experimental nc-AFM image, which agrees with the simulations. However, it shows a darker feature for the hydrogen saturated form at a moderate tip-sample separation (Supplementary Fig. 9c). These dark features originate from the hydrogen atom at the sp3 center, and are identified as bright protrusions at larger tip-sample separations.

(iii).The quinoid structure can be directly ruled out due to its complementary planar character, as can be seen in Supplementary Fig. 9h-9j.

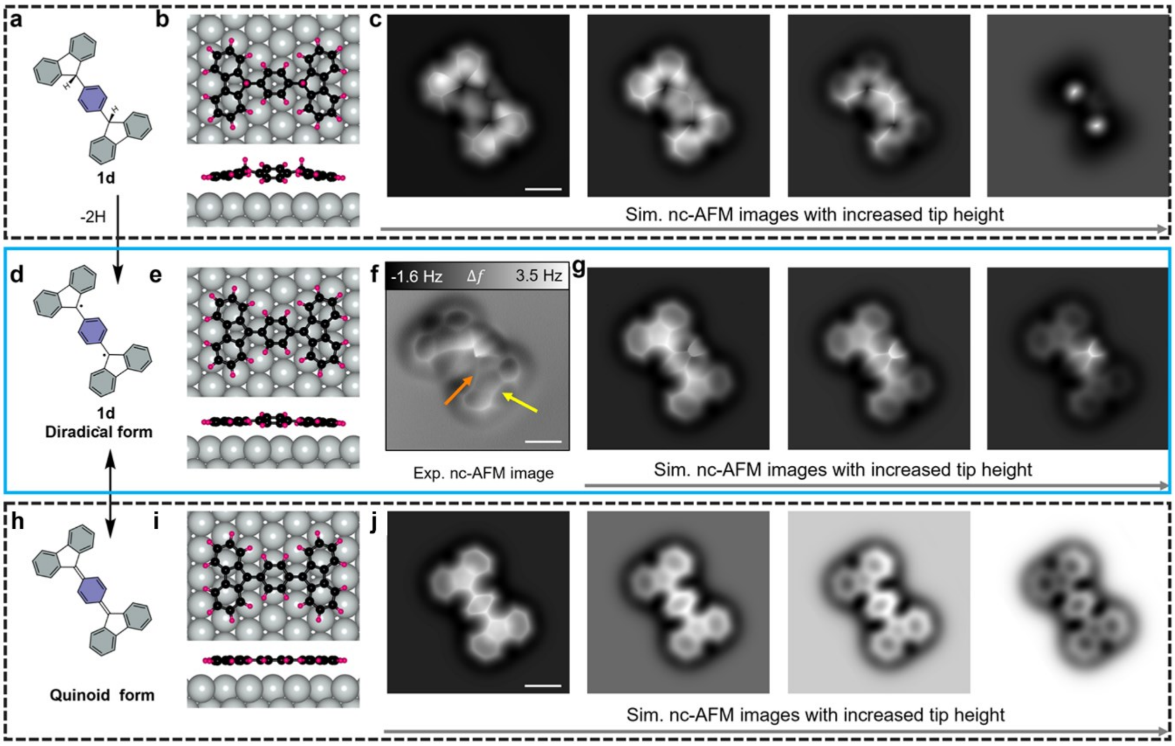

Supplementary Figure 9. Comparison of product 3 in different forms. a Chemical model of **3** in hydrogen saturated, **d** diradical and **h** quinoid form, respectively. **b**, **e**, **i** Optimized

adsorption configuration of the corresponding form and **c**, **g**, **j** the simulated nc-AFM image of intermediated **3** with increased tip height in **a**, **d**, **h**, respectively. **f**, experimental nc-AFM image (CO tip, $V_s = 2$ mV) of **3**. Scale bar: (**c**, **f**, **j**) 0.5 nm.

11. Partial cyclodehydrogenation products at increased temperatures.

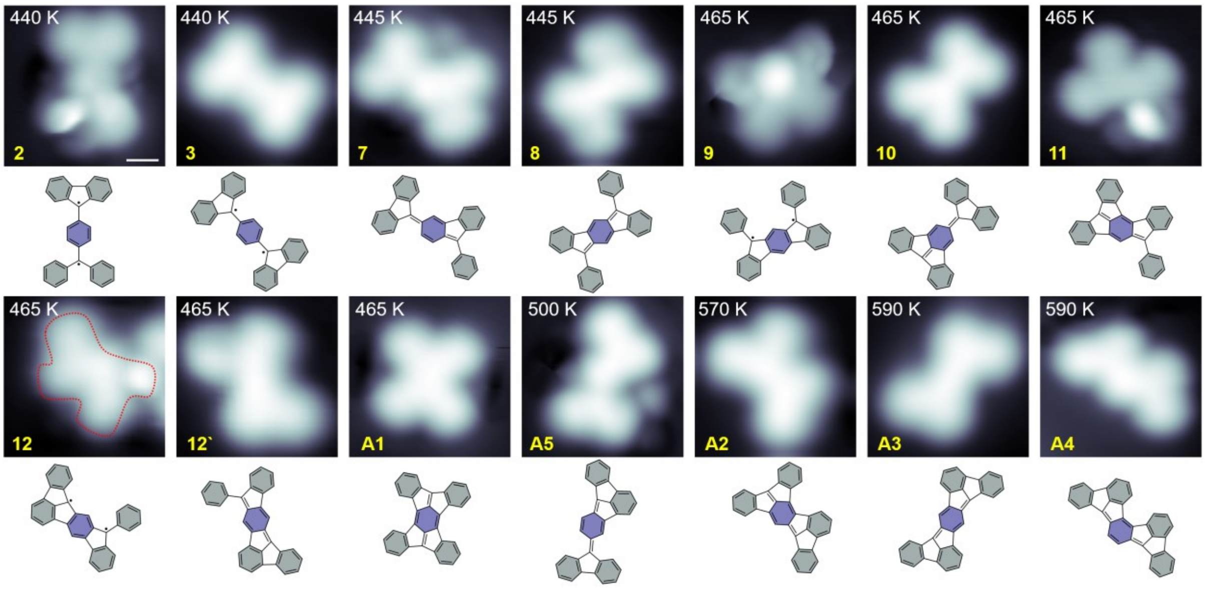

Supplementary Figure 10. STM topographies of cascaded cyclodehydrogenated products obtained at increased annealing temperatures. The corresponding chemical models are shown below each STM topography. Scale bars are all 0.5 nm. Scanning parameters: I = 50 pA, $V_s = 100$ mV for all STM topographic images. Note that **8** and **9** could not be obtained by a single step CDH from product **2**, implying the presence of **1d**, which is not captured directly in the experiment.

12. Additional BR-STM and nc-AFM images of products 3, 9, 13, 7, and 8.

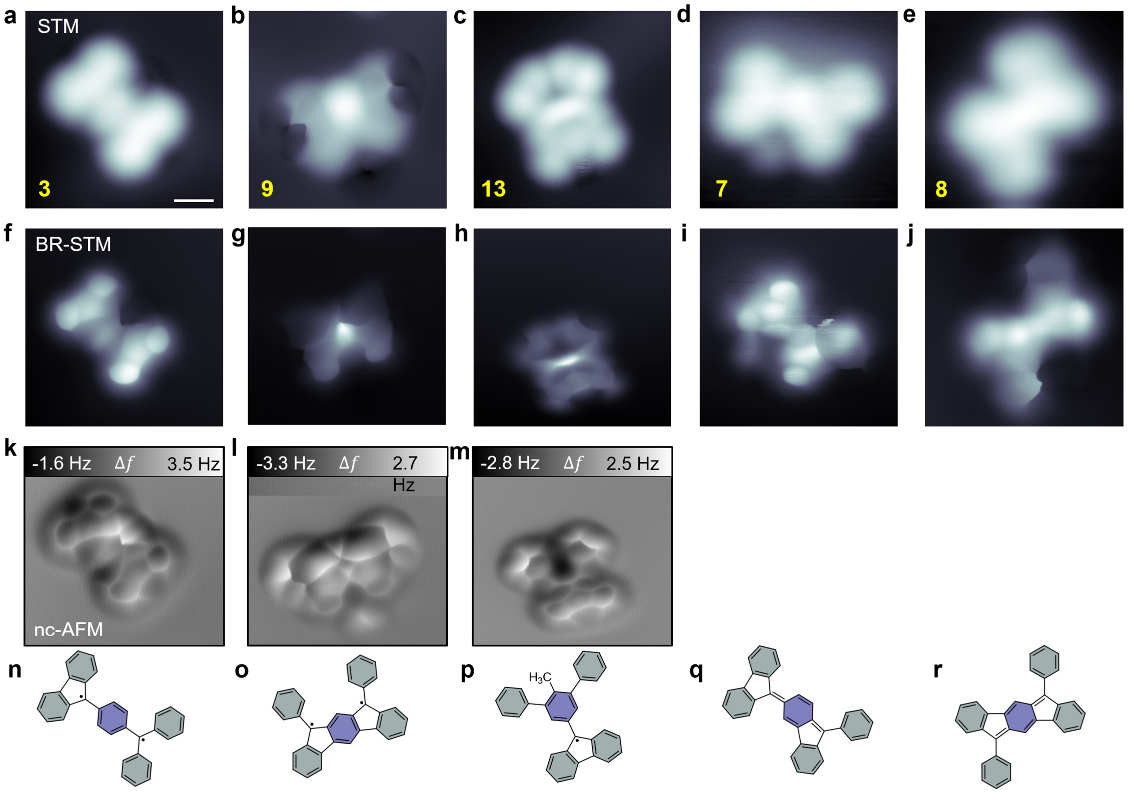

Supplementary Figure 11. Additional High-resolution STM and nc-AFM images of products 3, 9, 13, 7 and 8. a-e Constant current STM topographies (CO tip, I=50 pA, V_s=100 mV), **f-j** BR-STM images and **n-r** chemical models of the products **3, 9, 13, 7** and **8**. **k-m**, nc-AFM images (CO tip, V_s = 2 mV) correspond to panels **a-e**, respectively. Scale bar: 0.5 nm for all topographic images.

13. Fully cyclodehydrogenation products involve both phenyl group migration and direct C-C coupling.

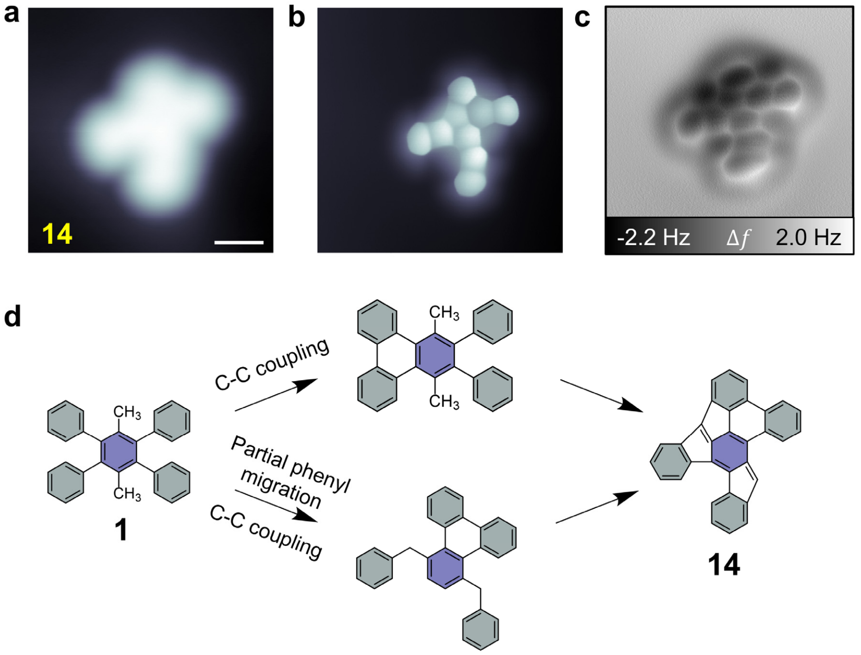

Supplementary Figure 12. Partially phenyl migrated CDH product 14. a Constant current STM topographies (CO tip, I = 50 pA, V_s = 100 mV). **b** BR-STM image and **c** nc-AFM image (CO tip, V_s = 2 mV) of partially phenyl migrated product **14**. **d** Possible mechanism to the formation of **14**. Scale bar: 0.5 nm.

14. Occasionally observed products 15 and A2`.

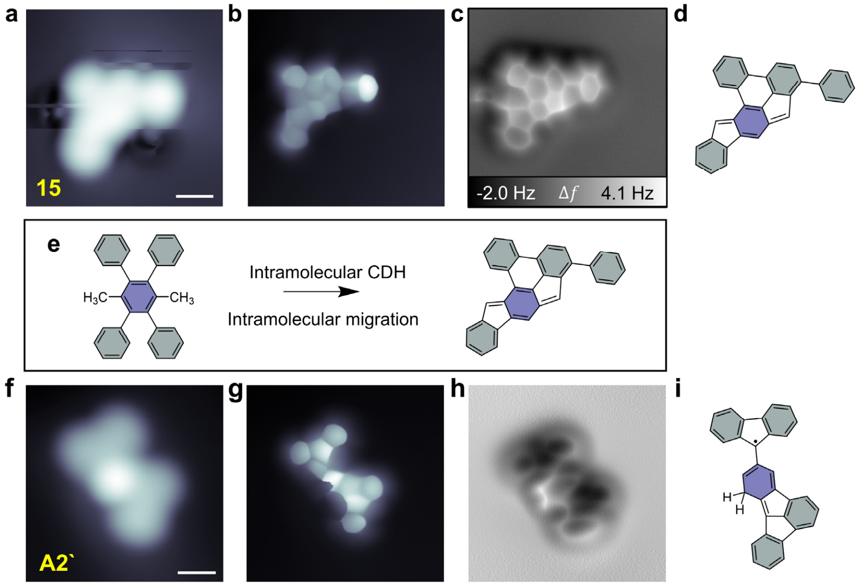

Supplementary Figure 13. Rarely observed intermediate products 15 and A2`. a, f** Constant current STM topographies (CO tip, I = 50 pA, $V_s$ = 100 mV) **b**, **g** Bond- resolved STM images and **c**, **h** nc-AFM images (CO tip, $V_s$ = 2 mV) of products **15** and A**2**`, respectively. **d, **i** The identified chemical structure of products **15** and A**2**`. **e** Scheme of the possible reaction mechanism to form **15**. Scale bar: 0.5 nm.

15. Chemical structure of product 10.

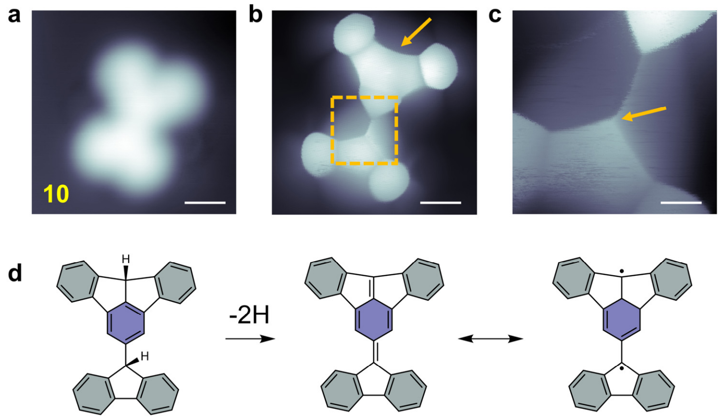

Supplementary Figure 14. Chemical structure of product 10. **a** Constant current STM image and **b** high-resolution BR-STM image and **c**, zoom-in BR-STM image of the area in (**b**) marked by an orange square. **d** Product **10** in different forms. Scale bar: (**a**) 4 Å; (**b**) 3 Å; (**c**) 1.2 Å.

16. Structural elucidation of cyclodehydrogenated products.

The cyclodehydrogenation intermediates **7**, **10** and **A2** can be simply regarded as hydrocarbons with a *p*-quinodimethane core (*p*-QDM)[1,2], as shown below in Supplementary Fig. 15. Considering that product **A2** gely present at 590 K (27%) as well as it can be found at a higher temperature, which is not in line with its highly reactive diradical character. We thus propose that product **A2** may have a closed-shell ground state with four sextets at the corners. This can be also evidenced by a shorter bond length between the fluorene and central benzene ring, as denoted by the yellow arrow in Supplementary Fig. 15c. Similar short bond length is also observed for **7** and **10**, as shown in Supplementary Fig. 15d and 15e. A dominant closed-shell ground state thus is also expected here.

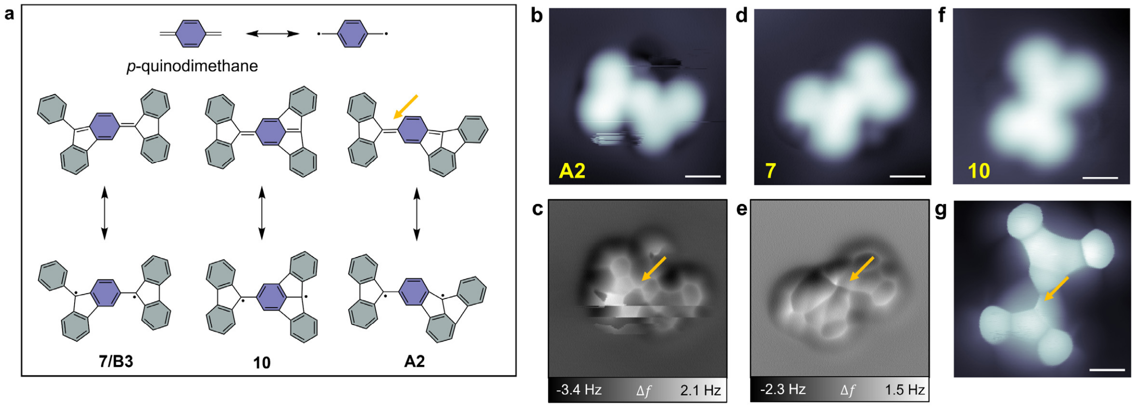

Supplementary Figure 15. Chemical structures of products A2, 7/B3 and 10. a Schematic illustration of possible canonical forms of considered structures. **b**, **d** STM and **c**, **e** nc-AFM images of products **A2** and **7**, respectively. **f** STM and **g** BR-STM images of product **10**. Scale bar: (**b-f**) 0.5 nm; (g)1.2 Å.

17. Chemical structural elucidation of cyclodehydrogenated products.

Although the additional formed five-membered ring may alter the conjugation, for the sake of simplicity, we here still regard them as indenofluorene derivatives. According to literature reports[3-5], indeno[1,2-b]fluorene has a canonical structure with diradical character y = 0.072, which means it has a dominant contribution of the closed-shell structure and indeno[2,1-b]fluorene has more open-shell character with y = 0.645. Note that product **11** can be also drawn as indeno[2,1-c]fluorene derivative (y = 0.021), as shown in Supplementary Fig. 16c, which also has a dominant closed-shell character. Based on the above discussion, we give the dominant resonance structure of these products in Supplementary Fig. 10.

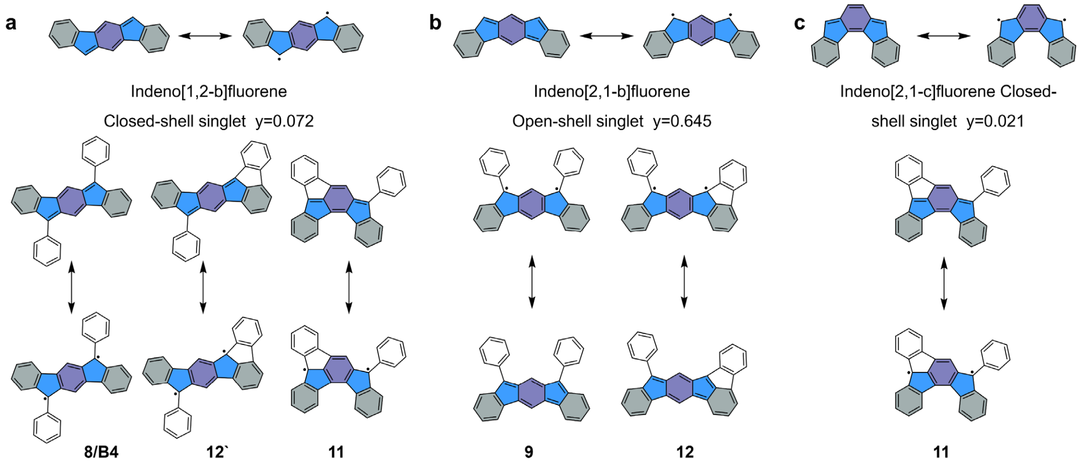

Supplementary Figure 16. Resonance structures of products 8/B4, 9, 11, 12 and 12`. a-c different indenofluorene regioisomers (top) and the corresponding classified products based on the indenofluorene core proposed (bottom).

18. Chemical structural elucidation of cyclodehydrogenated products A1, A2, A3, A4.

We assign the fully reacted products of nanographene **A1**-**A4** to the closed-shell canonical structure with four sextets as the BR-STM and their corresponding STS do not show any feature near the Fermi level, while the open-shell diradical form often gives rise to detectable features. Besides the STM-based measurements, in the nc-AFM images, the shorter bond length (yellow arrows) and brighter contrast (blue arrows) in Supplementary Fig. 17 indicate a higher bond order of these products, which explains their double bond character.

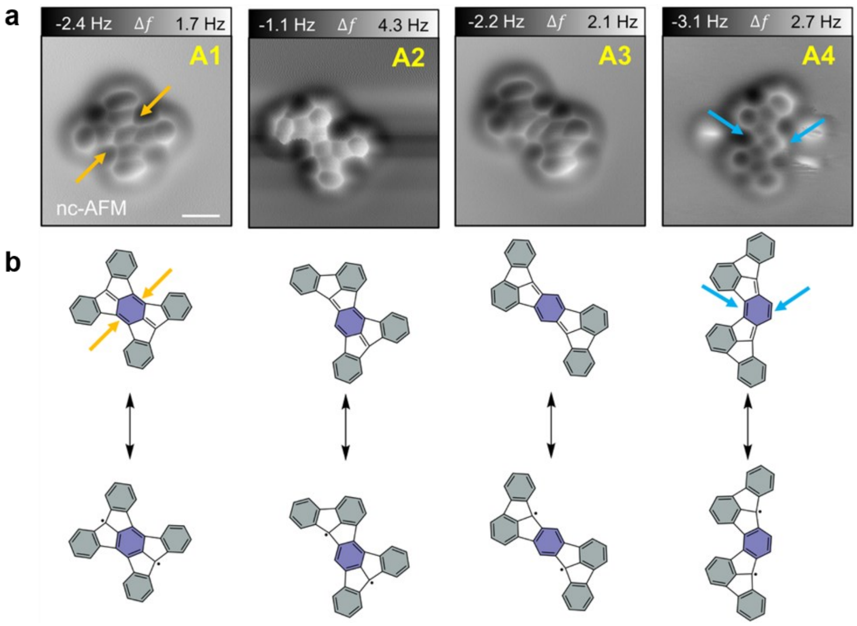

Supplementary Figure 17. Chemical structures of products A1-A4. **a** nc-AFM images of products **A1**-**A4** and **b** their chemical structures, respectively. Scale bar: 0.5 nm for all topographic images.

19. Detailed transformations from precursor 1 to 1a.

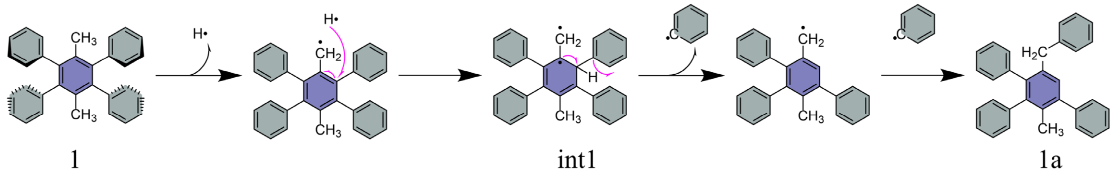

Supplementary Figure 18. The chemical transformation from 1 to 1a.

20. Comparison of three competing processes.

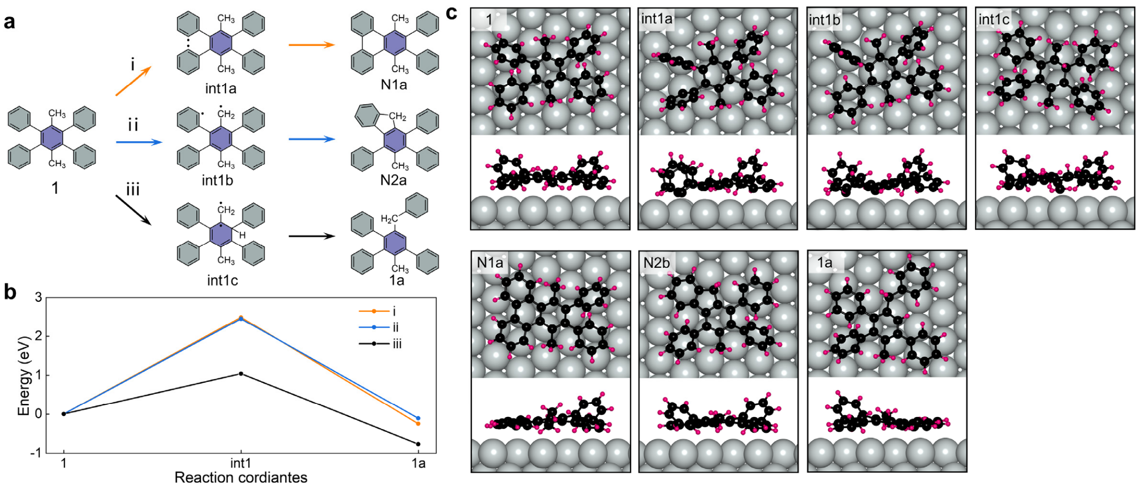

Supplementary Figure 19. Energy barrier comparison. a Schematic illustrations of three competing processes: i. cyclodehydrogenation of two adjacent phenyl groups; ii. C-C bond formation between a phenyl and a methyl group; iii. phenyl group migration. **b** Energy diagram comparing three competing processes. **c** Optimized configurations in **a**.

21. Stepwise phenyl groups migrations from 1 to 1d.

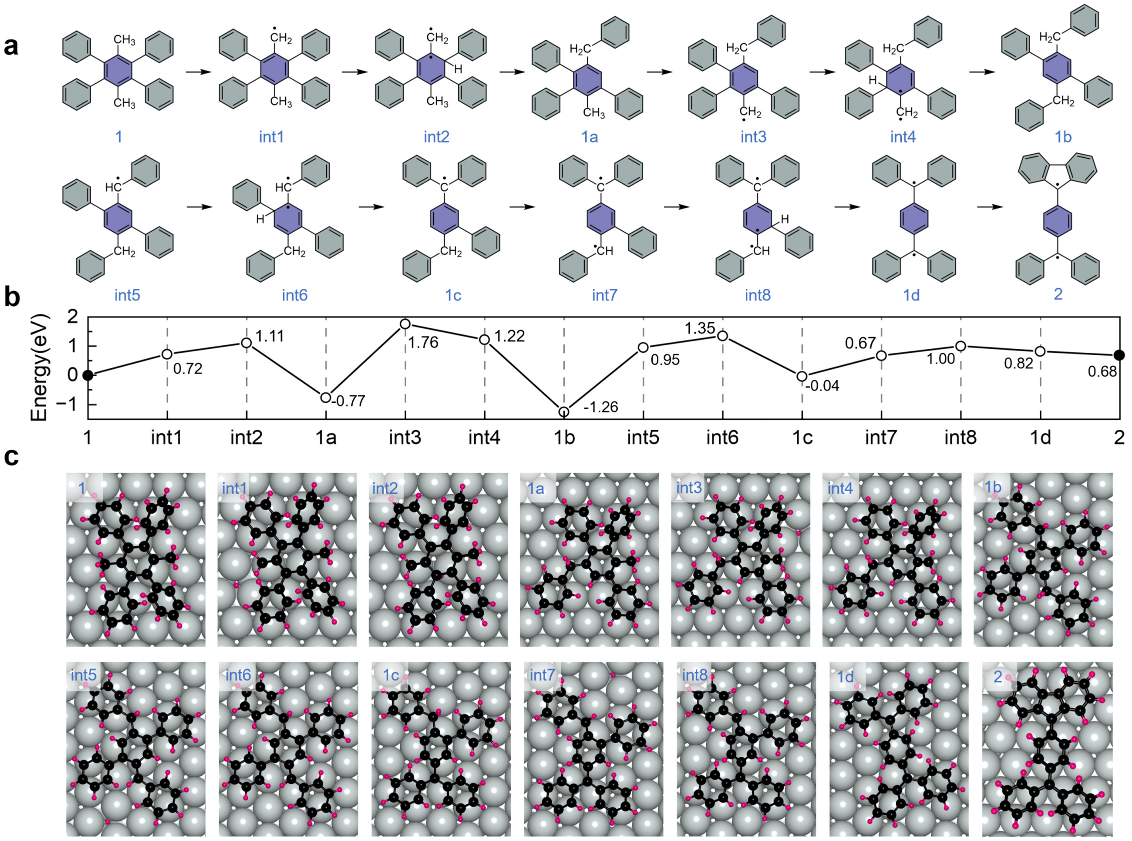

Supplementary Figure 20. DFT calculated energy diagram of the proposed phenyl migration. a Schematic illustration of the stepwise phenyl groups migrations from 1 to 1d. **b** Energy diagram of path B. **c** Optimized configurations in **a**. The experimentally observed species **1** and **2** are denoted with solid circles, while the intermediates 1a, 1b, 1c and 1d are denoted with hollow circles.

22. Reaction process analysis of DMTPB molecules on the Au(111) surface

In this paper, we analyzed the reaction process of DMTPB molecules on the Au(111) surface via the DFT total energies (enthalpic and entropic effects excluded) and Gibbs free energies (enthalpic and entropic effects included) associated with its adsorption. The DFT total energies obtained in VASP calculations are only electronic energies, which do not include some contributions from the internal energy, enthalpy, and entropy. The Gibbs free energies account for enthalpic and entropic effects at realistic temperatures when evaluating

the favorability of molecular adsorption on the Au(111) surface. Therefore, we need to calculate the additional terms contributing to the Gibbs free energy.

Firstly, the DFT electronic energies do not account for the zero point energy (ZPE) arising from atomic vibrations. The ZPE correction must be added to the electronic energy in order to calculate the internal energy. The total ZPE correction will be the sum of the individual energies arising from each vibrational mode, *K*, where there are 3n–5 vibrational modes in an n-atom linear molecule, 3n–6 in a nonlinear molecule, and 3n–3 in a crystal. The internal energy is calculated as:

$$E = E_{\text{elec}} + \text{ZPE} = E_{\text{elec}} + \sum_K \frac{1}{2} h\nu_K \quad (1)$$

where the final summation is the total ZPE correction.

At absolute zero and zero pressure, the internal energy and the enthalpy are equal. To account for enthalpy corrections at nonzero temperatures, the thermal corrections to the enthalpy can also be expressed as a function of the molecular partition function, where the correction from absolute zero to a specified temperature involves integrating the heat capacity at constant pressure:

$$H(T) - H(0) = \int_0^T C_p(T')dT' = k_B T^2 \frac{\partial ln\,(q)}{\partial T} + k_B T \quad (2)$$

Finally, the Gibbs free energy can be calculated by subtracting the contribution from entropy:

$$G = H - TS \quad (3)$$

Obtaining the Gibbs free energy completely from first principles, therefore, requires the calculation of the ZPE correction, the enthalpic temperature correction, and the entropic correction:

$$G = E_{\text{elec}} + \text{ZPE} + \int_0^T C_p(T')d\,T' - TS \quad (4)$$

When calculating the free energies of the clean slab and the slab with the adsorbed molecules, the vibrational component is the only contribution that must be considered, since

there are no longer any rotational or translational contributions to the free energy corrections. The total entropy is:

$$S = k_B\left[ln(q_v) + T\frac{\partial ln(q_v)}{\partial T} + 1\right] = k_B\left[\sum_K ln\left(\frac{1}{1-e^{-\Theta_{v,k}/T}}\right) + \sum_K \frac{\Theta_{v,k}}{T}\left(\frac{1}{e^{\Theta_{v,k}/T}-1} + 1\right)\right] \quad (5)$$

and the total enthalpic temperature correction is:

$$H_v(T) - H_v(0) = k_BT\left[\frac{T}{q_v}\frac{\partial q_v}{\partial T} + 1\right] = k_BT\left[\sum_K \frac{\Theta_{v,k}}{T}\left(\frac{1}{e^{\Theta_{v,k}/T}-1} + 1\right)\right] \quad (6)$$

Zero-point energy (ZPE), enthalpy, and entropy contributions to free energies of DMTPB molecules on the Au(111) surface at 440 K were calculated from vibrational modes of surface species, which were computed with the finite difference approach as implemented in the VaspGibbs code.[6] At 440 K where the reactions take place, the removed hydrogen atoms will combine and desorb as H_2. Therefore, the energy of the removed hydrogen atom can be treated as $\frac{1}{2}E(H_2)$. The Gibbs free energies of H_2 molecules at 440 K which Zero-point energy (ZPE), enthalpy, and entropy contributions are included were computed with the finite difference approach as implemented in the VaspGibbs code. The energy difference between the Gibbs free energy of H_2 and the DFT total energy of H_2 is 0.10 eV. The thermal correction ΔE(H) of the removed hydrogen can be treated as 0.05 eV per H atom. The ZPE, entropic, and enthalpic contributions for the adsorbed slabs in Paths 1-4 at 440 K are listed in the Supplementary Tables 2-5.

Supplementary Table 2. ZPE, entropic, enthalpic temperature contributions and relative Gibbs free energies for the adsorbed slabs in Path 1 at 440 K. The DFT total energy E_{elec} and Gibbs free energy G of the experimentally observed species **1** are set as zero points respectively.

	ZPE (eV)	S (eV/K)	-TS (eV)	$\int_0^T C_p(T')dT'$ (eV)	ZPE+ $\int_0^T C_p(T')dT'$-TS (eV)	E_{elec} (eV)	E_{elec}+ZPE+ $\int_0^T C_p(T')dT'$- $TS+\frac{n}{2}\Delta E$(H) (eV)	G (eV)
1	13.72	5.22×10^{-2}	-22.95	8.62	-0.61	0	-0.61	0
1'	13.10	5.20×10^{-2}	-22.87	8.59	-1.17	2.10	1.03	1.64
1a	13.11	5.21×10^{-2}	-22.91	8.60	-1.20	1.20	0.1	0.71
1b	13.15	5.16×10^{-2}	-22.71	8.55	-1.00	0.27	-0.63	-0.02

1c	13.16	5.20×10^{-2}	-22.88	8.58	-1.06	0.62	-0.34	0.27
1d	13.18	5.19×10^{-2}	-22.84	8.57	-1.04	0.82	-0.12	0.49
2	12.58	5.14×10^{-2}	-22.62	8.49	-1.53	0.68	-0.75	-0.14

Supplementary Table 3. ZPE, entropic, enthalpic temperature contributions and relative Gibbs free energies for the adsorbed slabs in Path 2 at 440 K. The DFT total energy E_{elec} and Gibbs free energy G of the experimentally observed species **1** are set as zero points respectively.

	ZPE (eV)	S(eV/K)	-TS (eV)	$\int_0^T C_p(T')dT'$ (eV)	ZPE+ $\int_0^T C_p(T')dT'$-TS (eV)	E_{elec} (eV)	E_{elec}+ZPE+ $\int_0^T C_p(T')dT'$-TS+$\frac{n}{2}\Delta E$(H) (eV)	G (eV)
1	13.72	5.22×10^{-2}	-22.95	8.62	-0.61	0	-0.61	0
1a	13.43	5.187×10^{-2}	-22.83	8.564	-0.83	-0.26	-1.04	-0.43
1b	13.15	5.16×10^{-2}	-22.71	8.55	-1.00	0.27	-0.63	-0.02
1c	13.16	5.20×10^{-2}	-22.88	8.58	-1.06	0.62	-0.34	0.27
1d	13.18	5.19×10^{-2}	-22.84	8.57	-1.04	0.82	-0.12	0.49
2	12.58	5.14×10^{-2}	-22.62	8.49	-1.53	0.68	-0.75	-0.14

Supplementary Table 4. ZPE, entropic, enthalpic temperature contributions and relative Gibbs free energies for the adsorbed slabs in Path 3 at 440 K. The DFT total energy E_{elec} and Gibbs free energy G of the experimentally observed species **1** are set as zero points respectively.

	ZPE (eV)	S (eV/K)	-TS (eV)	$\int_0^T C_p(T')dT'$ (eV)	ZPE+ $\int_0^T C_p(T')dT'$-TS (eV)	E_{elec} (eV)	E_{elec}+ZPE+ $\int_0^T C_p(T')dT'$-TS (eV)+$\frac{n}{2}\Delta E$(H)	G (eV)
1	13.72	5.22×10^{-2}	-22.95	8.62	-0.61	0	-0.61	0
1a	13.77	5.20×10^{-2}	-22.87	8.61	-0.49	-0.77	-1.26	-0.65
1b	13.76	5.14×10^{-2}	-22.63	8.54	-0.33	-1.26	-1.59	-0.98
1c	13.43	5.20×10^{-2}	-22.88	8.57	-0.90	-0.04	-0.89	-0.28
1d	13.18	5.19×10^{-2}	-22.84	8.57	-1.04	0.82	-0.12	0.49
2	12.58	5.14×10^{-2}	-22.62	8.49	-1.53	0.68	-0.75	-0.14

Supplementary Table 5. ZPE, entropic, enthalpic temperature contributions and relative Gibbs free energies for the adsorbed slabs in Path 4 at 440 K. The DFT total energy E_{elec} and

Gibbs free energy G of the experimentally observed species **1** are set as zero points respectively.

	ZPE (eV)	S (eV/K)	-TS (eV)	$\int_0^T C_p(T')dT'$ (eV)	ZPE+ $\int_0^T C_p(T')dT'$-TS (eV)	E_{elec} (eV)	E_{elec}+ZPE+ $\int_0^T C_p(T')dT'$- TS+$\frac{n}{2}\Delta E$(H) (eV)	G (eV)
1	13.72	5.22×10^{-2}	-22.95	8.62	-0.61	0	-0.61	0
1a	13.77	5.20×10^{-2}	-22.87	8.61	-0.49	-0.77	-1.26	-0.65
1b	13.76	5.14×10^{-2}	-22.63	8.54	-0.33	-1.26	-1.59	-0.98
1b’	13.13	5.17×10^{-2}	-22.76	8.55	-1.07	0.19	-0.78	-0.17
1c	13.16	5.20×10^{-2}	-22.88	8.58	-1.06	0.62	-0.34	0.27
1d	13.18	5.19×10^{-2}	-22.84	8.57	-1.04	0.82	-0.12	0.49
2	12.58	5.14×10^{-2}	-22.62	8.49	-1.53	0.68	-0.75	-0.14

Accordingly, energy diagrams with and without enthalpic and entropic effects for the stepwise phenyl groups migrations in path 1 (dehydrogenations precede phenyl groups migrations), path 2 (dehydrogenations occur during 1-1a and 1a-1b), path 3 (dehydrogenations occur during 1b-1c and 1c-1d) and path 4 (dehydrogenations occur after two phenyl groups migrations) are compared respectively, as shown in Supplementary Figures 21-24.

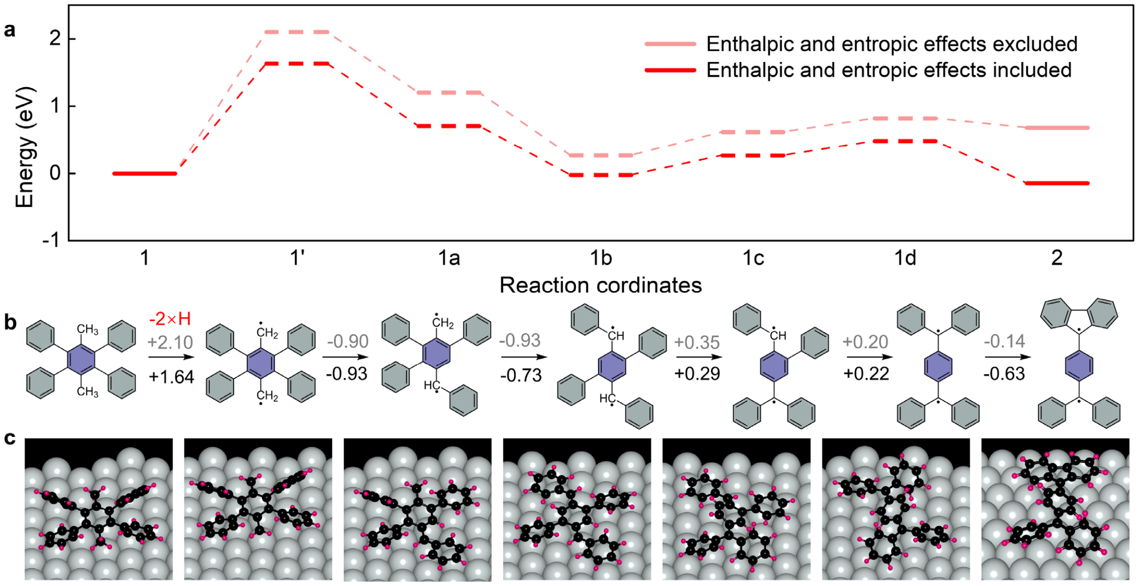

Supplementary Figure 21. Energy diagram of the potential stepwise migration reaction (path 1). (a) Energy diagram with (dark) and without (light) including enthalpic and entropic

effects for the stepwise phenyl groups migrations in path 1: dehydrogenations precede phenyl groups migrations. (b) Schematic illustration of the stepwise phenyl groups migrations from **1** to **2**. (c) Optimized configurations in (a). The experimentally observed species **1** and **2** are denoted with solid lines, while the intermediates **1a, 1b, 1c** and **1d** are denoted with dashed lines.

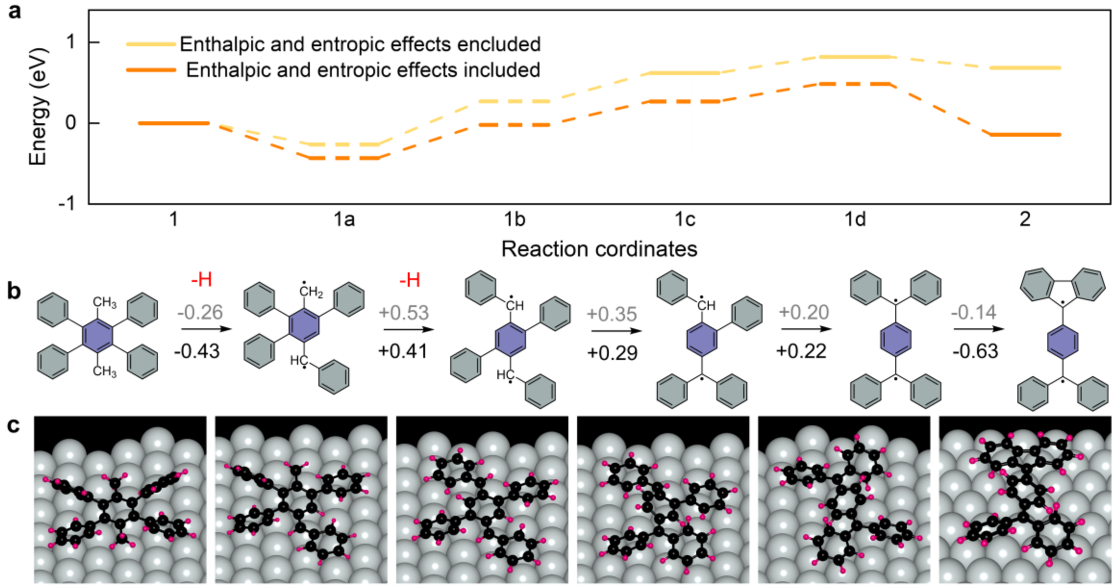

Supplementary Figure 22. Energy diagram of the potential stepwise migration reaction (path 2). (a) Energy diagram with (dark) and without (light) including enthalpic and entropic effects for the stepwise phenyl groups migrations in path 2: dehydrogenations occur during **1-1a** and **1a-1b**. (b) Schematic illustration of the stepwise phenyl group migrations from **1 to 2.** (c) Optimized configurations in (a). The experimentally observed species **1** and **2** are denoted with solid lines, while the intermediates **1a**, **1b**, **1c** and **1d** are denoted with dashed lines.

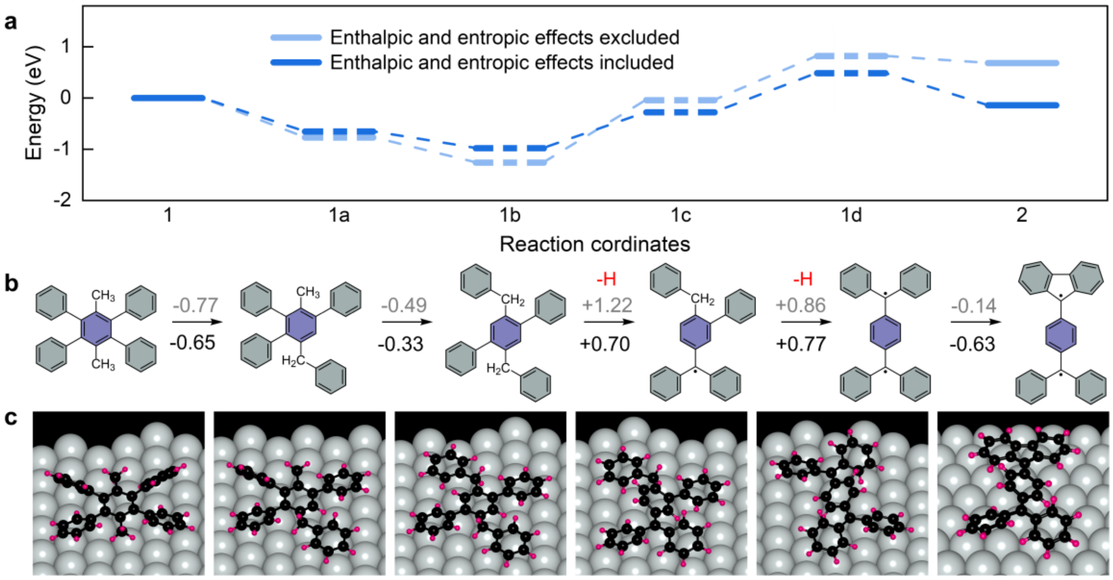

Supplementary Figure 23. Energy diagram of the potential stepwise migration reaction (path 3). (a) Energy diagram with (dark) and without (light) including enthalpic and entropic effects for the stepwise phenyl groups migrations in path 3: dehydrogenations occur during **1b-1c** and **1c-1d**. (b) Schematic illustration of the stepwise phenyl group migrations from 1 to 1d. (c) Optimized configurations in (a). The experimentally observed species 1 and 2 are denoted with solid lines, while the intermediates 1a, 1b, 1c and 1d are denoted with dashed lines.

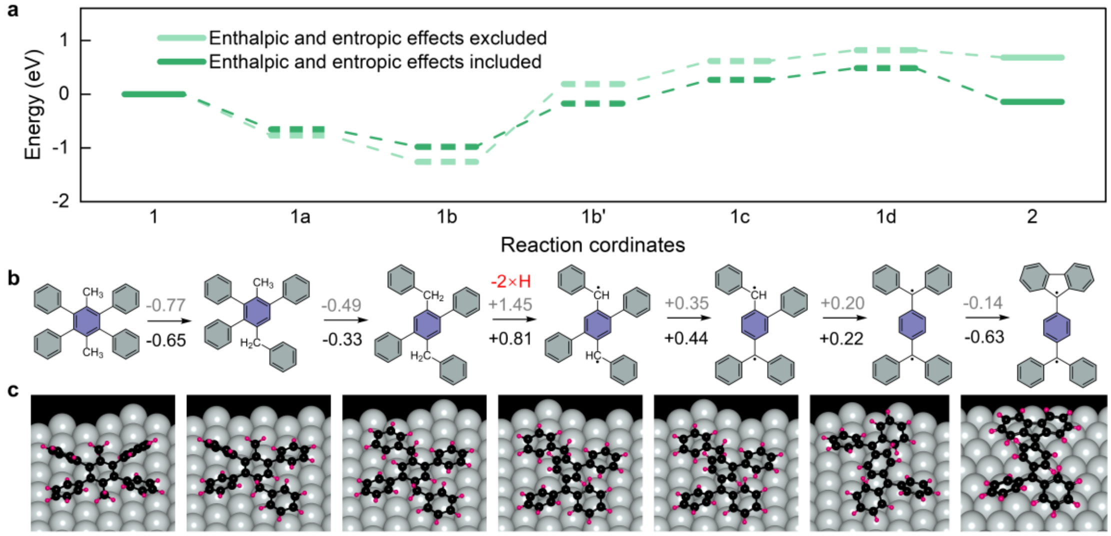

Supplementary Figure 24. Energy diagram of the potential stepwise migration reaction (path 4). (a) Energy diagram with (dark) and without (light) including enthalpic and entropic effects for the stepwise phenyl groups migrations in path 4: dehydrogenations occur after two phenyl groups migrations. (b) Schematic illustration of the stepwise phenyl group migrations

from **1** to **2**. (c) Optimized configurations in (a). The experimentally observed species **1** and **2** are denoted with solid lines, while the intermediates **1a, 1b, 1c** and **1d** are denoted with dashed lines.

As listed in Supplementary Tables 2-5 or shown in Supplementary Figures 21-25, there are energy differences between the DFT total energies (enthalpic and entropic effects excluded) and Gibbs free energies (enthalpic and entropic effects included) for each step. And when hydrogen atoms are removed from the system, the energy differences are relatively large.

Considering that the extra hydrogen atoms are going to get away, endothermic reactions are possible to happen. As shown in Supplementary Figs. 21-24, four possibilities were considered: path 1 that dehydrogenations precede phenyl groups migrations (Supplementary Fig. 21); path 2 that dehydrogenations occur during **1-1a** and **1a-1b** (Supplementary Fig. 22); path 3 that dehydrogenations occur during **1b-1c** and **1c-1d** (Supplementary Fig. 23); path 4 that dehydrogenations occur after the migrations of two phenyl groups (Supplementary Fig. 24). Energy diagram comparing all four plausible reaction mechanisms that lead from **1** to **2** are shown in Supplementary Fig. 25, which reveals that path 3 (dehydrogenating during 1b-1c and 1c-1d) is the energetic favorable reaction path.

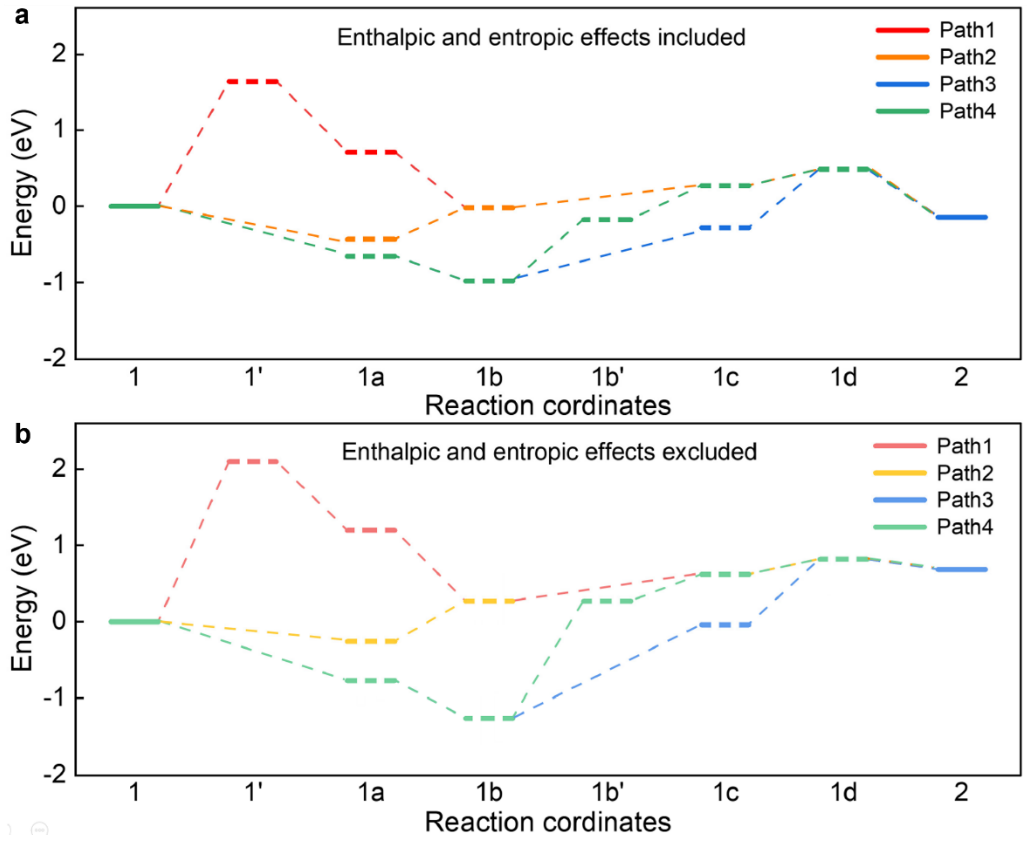

Supplementary Figure 25. Energy diagram comparing all four plausible reaction mechanisms that lead from 1 to 2. Energy diagram comparing all four plausible reaction mechanisms **a)** with and **b)** without including enthalpic and entropic effects. Path 1: dehydrogenations precede phenyl groups migrations. Path 2: dehydrogenations occur during **1-1a** and **1a-1b**. Path 3: dehydrogenations occur during **1b-1c** and **1c-1d**. Path 4: dehydrogenations occur after two phenyl groups migrations. The experimentally observed species 1 and 2 are denoted with solid lines, while the intermediates **1a, 1b, 1c** and **1d** are denoted with dashed lines.

23. DMTPB self-assembly on Cu(111).

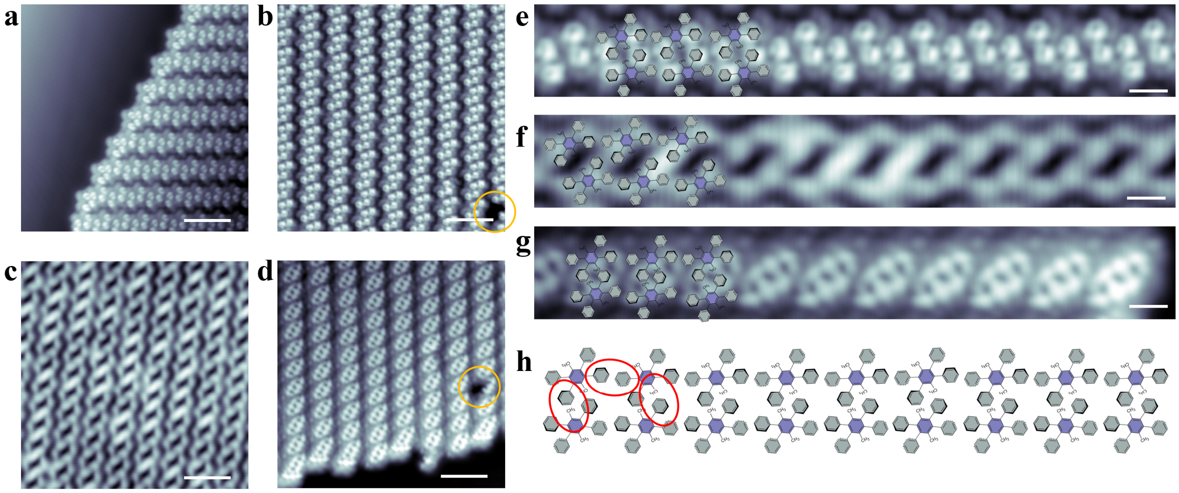

Supplementary Figure 26. STM topographies of DMTPB self-assembly after deposited on Cu(111) kept at room temperature. a-d STM topographies of self-assembly obtained under different tunneling conditions. The light-yellow circles in b and d denoted defects produced by picking up a monomer by tip manipulation. **e-g** A single chain cut from (**b-d**), respectively. **h** Structural model corresponds to (**e-f**). The red ellipses denote the CH···π and π···π interactions. Scale bar: (**a-d**) 4 nm; (**e-f**) 1 nm. Scanning parameters: (**a**) I = 80 pA, V_s = 1 V; (**b**) I = 50 pA, V_s = 1.2 V; (**c**) I = 50 pA, V_s = 0.8 V; (**d**) I = 50 pA, V_s = -2 V.

24. Supplementary Table 6. Statistics of the major products after annealing the DMTPB self-assembly on Cu(111) at 420 K.

Supplementary table 6: Statistics of the major products after annealing the DMTPB self-assembly on Cu(111) to 420 K.

Products	B1	B2	B3	B4	
Counts	53	6	50	34	143
Yield	37%	4%	35%	24%	100%

25. Identification of monomer B1 and B5 on Cu(111) surface.

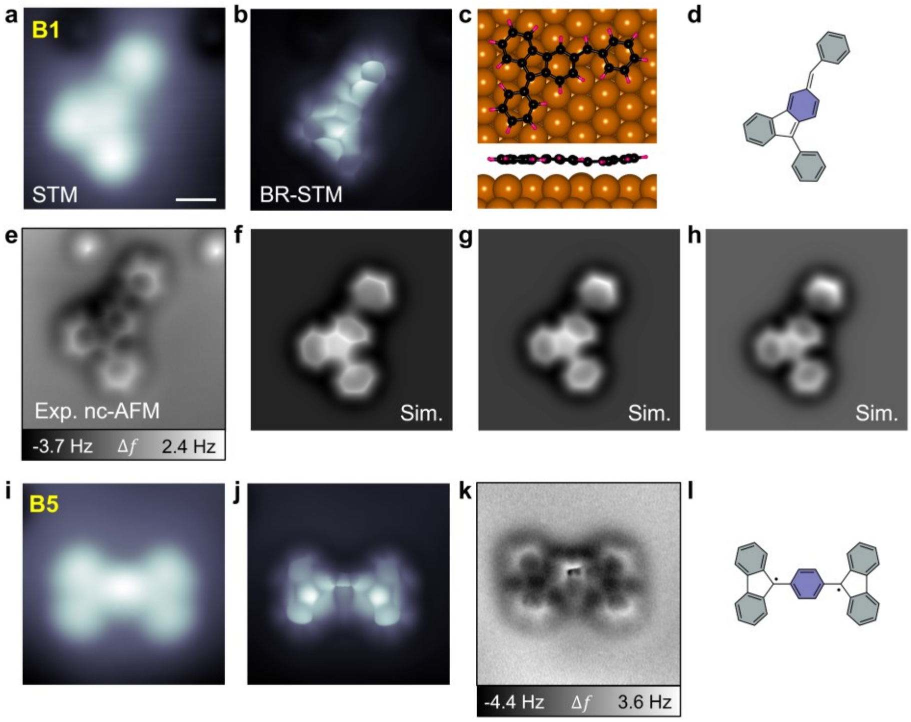

Supplementary Figure 27. Identification of monomer B1 and B5 on Cu(111) surface. a, **i** STM topographies. **b**, **j** BR-STM images. **c** top view and side view of optimized monomer **B1** on Cu(111). **e**, **k** nc-AFM images and **d**, **l** identified chemical structure of **B1** and **B5** on Cu(111), respectively. **f-h** Simulated nc-AFM images of **B1**. Scanning parameters: (**a**, **i**) I = 50 pA, V_s = 100 mV. Scale bar: (**a**, **b**, **e-k**) 0.5 nm.

26. Additional High-resolution STM topographies of products on Cu (111) surface.

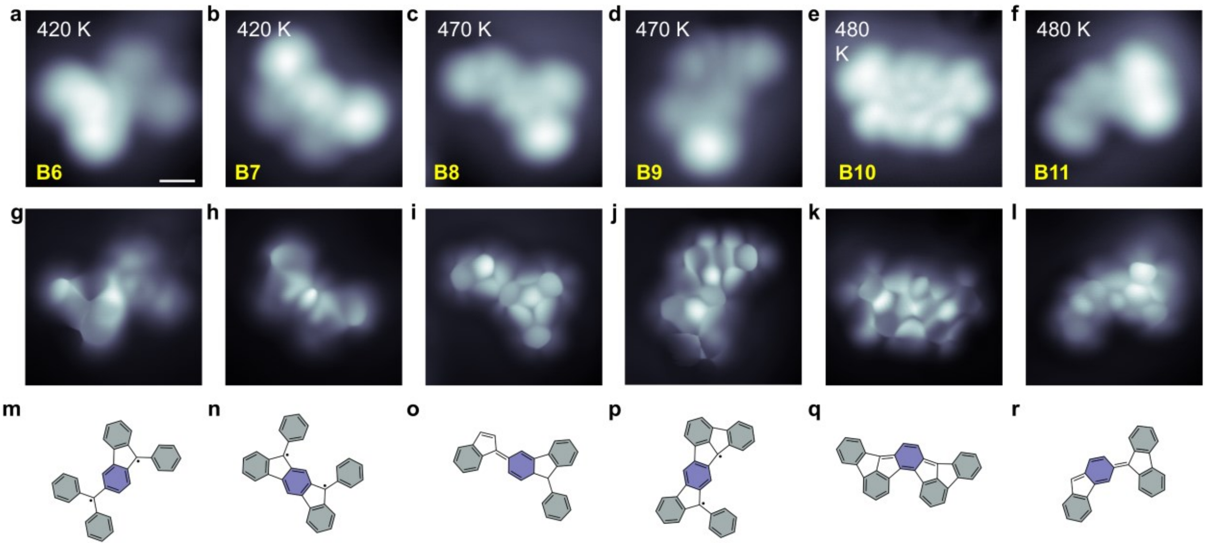

Supplementary Figure 28. Monomer products obtained after increasing annealing temperature. **a-f** STM topographies of different products. **g-l** BR-STM images of products corresponding to (**a-f**), respectively. **m-r** The identified molecular structures of six products corresponding to panels (**g–l**), respectively. Scanning parameters: (**a-f**) I = 50 pA, V_s = 100 mV; (**g-l**) V_s = 2 mV. Scale bar: 0.4 nm.

27. DMTPB on Ag(110) surface after RT deposition.

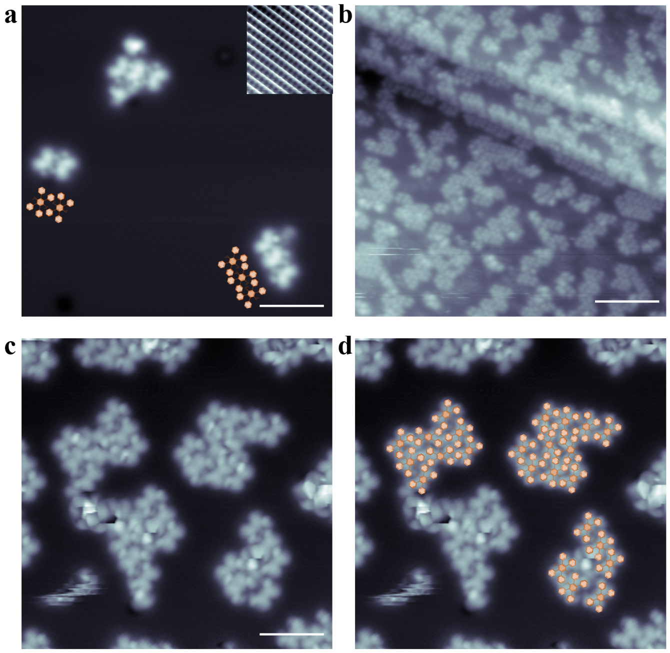

Supplementary Figure 29. DMTPB on Ag(110) after RT deposition. **a**, **b** STM topographies of DMTPB on Ag(110) at a low and high coverage, respectively. The inset shows the atomic resolution of Ag(110) surface. **c** Closed-up STM topography of DMTPB on Ag(110) at a high coverage. **d** The same STM topography with (**c**) with partially overlaid molecular models. Scale bar: (**a**) 3.6 nm; (**b**) 10 nm; (**c**) 3 nm. Scanning parameters: (**a**) I = 20 pA, V_s = 0.1 V; (**b**, **c**) I = 20 pA, V_s = 1 V.

28. Identified monomers on the Ag(110) surface after annealing to 460 K.

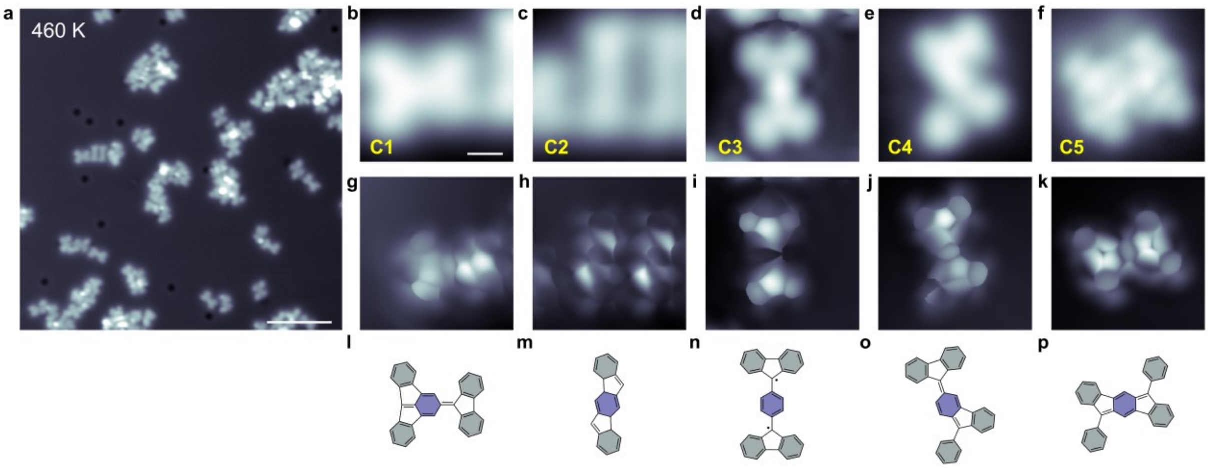

Supplementary Figure 30. Monomer products formed on the Ag(110) surface. a Large scale STM topography of different products after thermal annealing of the DMTPB self-assembly on Ag(110) to 460 K. **b-f** STM topographies of major monomer products. BR-STM images of monomer products corresponding to (**b-f**), respectively. **l-p** the identified molecular structures of five products corresponding to panels (**g**–**k**), respectively. Scale bar: (**a**) 6 nm; (**b-k**) 0.4 nm. Scanning parameters: (**a**) I = 20 pA, V_s = 100 mV; (**b-f**) I = 50 pA, V_s = 100 mV; (**g-k**) V_s = 2 mV.

Supplementary references

1. Tan, G. & Wang, X. Isolable bis (triarylamine) dications: analogues of Thiele's, Chichibabin's, and Muller's hydrocarbons. *Acc. Chem. Res.* **50**, 1997-2006 (2017).
2. Zeng, Z. *et al.* Pushing extended p-quinodimethanes to the limit: stable tetracyano-oligo (N-annulated perylene) quinodimethanes with tunable ground states. *J. Am. Chem. Soc.* **135**, 6363-6371 (2013).
3. Fukuda, K. Nagami, T. Fujiyoshi, J. Y. & Nakano, M. Interplay between open-shell character, aromaticity, and second hyperpolarizabilities in indenofluorenes. *J. Phys. Chem. A* **119**, 10620-10627 (2015).
4. Di Giovannantonio, M. *et al.* On-surface synthesis of antiaromatic and open-shell indeno [2, 1-b] fluorene polymers and their lateral fusion into porous ribbons. *J. Am. Chem. Soc.* **141**, 12346-12354 (2019).
5. Rudebusch, G. E. *et al.* Diindeno-fusion of an anthracene as a design strategy for stable organic biradicals. *Nat. Chem.* **8,** 753-759 (2016).
6. Bendavid, L. I., & Carter, E. A. CO_2 adsorption on Cu_2O (111): a DFT+ U and DFT-D study. *J. Phys. Chem. C* **117**, 26048-26059 (2013).
